# Supplementary material for: MDM2 functions as a timer reporting the length of mitosis
Source: Nat Cell Biol. 2025 Jan 9;27(2):262–72. doi: 10.1038/s41556-024-01592-8 (PMC11821534; doi:10.1038/s41556-024-01592-8)
Supplement: Supplementary file 1 — Reporting Summary [file 41556_2024_1592_MOESM1_ESM.pdf]

nature portfolio

Corresponding author(s): Francis A. Barr

Last updated by author(s): Nov 19, 2024

Reporting Summary

Nature Portfolio wishes to improve the reproducibility of the work that we publish. This form provides structure for consistency and transparency in reporting. For further information on Nature Portfolio policies, see our Editorial Policies and the Editorial Policy Checklist.

Statistics

For all statistical analyses, confirm that the following items are present in the figure legend, table legend, main text, or Methods section.

n/a	Confirmed	
☐	☒	The exact sample size (*n*) for each experimental group/condition, given as a discrete number and unit of measurement
☐	☒	A statement on whether measurements were taken from distinct samples or whether the same sample was measured repeatedly
☐	☒	The statistical test(s) used AND whether they are one- or two-sided<br>*Only common tests should be described solely by name; describe more complex techniques in the Methods section.*
☒	☐	A description of all covariates tested
☒	☐	A description of any assumptions or corrections, such as tests of normality and adjustment for multiple comparisons
☐	☒	A full description of the statistical parameters including central tendency (e.g. means) or other basic estimates (e.g. regression coefficient) AND variation (e.g. standard deviation) or associated estimates of uncertainty (e.g. confidence intervals)
☐	☒	For null hypothesis testing, the test statistic (e.g. *F*, *t*, *r*) with confidence intervals, effect sizes, degrees of freedom and *P* value noted<br>*Give P values as exact values whenever suitable.*
☒	☐	For Bayesian analysis, information on the choice of priors and Markov chain Monte Carlo settings
☒	☐	For hierarchical and complex designs, identification of the appropriate level for tests and full reporting of outcomes
☒	☐	Estimates of effect sizes (e.g. Cohen's *d*, Pearson's *r*), indicating how they were calculated

Our web collection on statistics for biologists contains articles on many of the points above.

Software and code

Policy information about availability of computer code

Data collection	Plate imaging: Bio-Rad Gel Doc XR+ imaging system (Bio-Rad, 1708195EDU);<br>Immunofluorescence: MetaMorph 7.5 imaging software (Molecular Devices);<br>Live cell imaging: Volocity 6 software (PerkinElmer)
Data analysis	Statistical analysis: GraphPad Prism v.10.3.0 (GraphPad Software);<br>Image analysis: Fiji/Image J (v.2.14.0) software (National Institutes of Health); Image Lab 6 (Bio-Rad)

For manuscripts utilizing custom algorithms or software that are central to the research but not yet described in published literature, software must be made available to editors and reviewers. We strongly encourage code deposition in a community repository (e.g. GitHub). See the Nature Portfolio guidelines for submitting code & software for further information.

Data

Policy information about availability of data

All manuscripts must include a data availability statement. This statement should provide the following information, where applicable:

- Accession codes, unique identifiers, or web links for publicly available datasets
- A description of any restrictions on data availability
- For clinical datasets or third party data, please ensure that the statement adheres to our policy

All source data (numerical data and uncropped blots) relating to the manuscript are provided with this manuscript.

Research involving human participants, their data, or biological material

Policy information about studies with human participants or human data. See also policy information about sex, gender (identity/presentation), and sexual orientation and race, ethnicity and racism.

Reporting on sex and gender	N/A
Reporting on race, ethnicity, or other socially relevant groupings	N/A
Population characteristics	N/A
Recruitment	N/A
Ethics oversight	N/A

Note that full information on the approval of the study protocol must also be provided in the manuscript.

Field-specific reporting

Please select the one below that is the best fit for your research. If you are not sure, read the appropriate sections before making your selection.

☒ Life sciences ☐ Behavioural & social sciences ☐ Ecological, evolutionary & environmental sciences

For a reference copy of the document with all sections, see nature.com/documents/nr-reporting-summary-flat.pdf

Life sciences study design

All studies must disclose on these points even when the disclosure is negative.

Sample size	No statistical methods were used to pre-determine the experimental sample sizes. Sample size was chosen based on accepted standards in the field, as outlined by Lord, S.J. et al, (2020), "SuperPlots: Communicating reproducibility and variability in cell biology", JCB, and Zweifach, A. (2024), "Determining how many cells to average for statistical testing of microscopy experiments", JCB. The exact sample size of the associated experiments are described in the Figure legends.
Data exclusions	No data were excluded from the analyses.
Replication	The exact number of independent replicates/experiments, at least 3, are indicated in the corresponding figures or their legends. All effects observed were both reproducible and penetrant.
Randomization	All experiments were performed with molecular biological techniques. Experiments were performed with independent replicates and populations of manipulated cell lines for cell culture-based experiments. Microscopy images in each sample were acquired from randomly selected areas.
Blinding	The investigators were not blinded during data collection or analysis. Image and Western blot data was analysed systematically using ImageJ/FIJI with stastical analysis in GraphPad Prism, and we determined no blinding was required.

Reporting for specific materials, systems and methods

We require information from authors about some types of materials, experimental systems and methods used in many studies. Here, indicate whether each material, system or method listed is relevant to your study. If you are not sure if a list item applies to your research, read the appropriate section before selecting a response.

Materials & experimental systems

n/a	Involved in the study	
☐	☒	Antibodies
☐	☒	Eukaryotic cell lines
☒	☐	Palaeontology and archaeology
☒	☐	Animals and other organisms
☒	☐	Clinical data
☒	☐	Dual use research of concern
☒	☐	Plants

Methods

n/a	Involved in the study	
☒	☐	ChIP-seq
☒	☐	Flow cytometry
☒	☐	MRI-based neuroimaging

Antibodies

Antibodies used	Supplementary Method Table 1 describes all antibodies used for WB, IF and IP in this study.
Validation	All commercial primary antibodies were validated by the supplier using human cells, in multiple independent previous studies, and in this study (please see below):<br>MDM2 (IF2) Millipore mAb OP46 Mouse - validated by siRNA - not included in the manuscript but available on request. Also validated by MD-224 PROTAC treatment (Figure 4b,5g; Extended Data Figure 7b-f).<br>MDM2 (SMP14) Abcam mAb Ab3110 Mouse - validated by siRNA - not included in the manuscript but available on request.<br>MDM2 (D1V2Z) CST mAb 86934 Rabbit - validated by siRNA - not included in the manuscript but available on request.<br>p53 (DO-7) CST mAb 48818 Mouse - validated by KO (Figure 4b).<br>p53 (7F5) CST mAb 2527 Rabbit - validated by KO - not included in the manuscript but available on request.<br>p21 (12D1) CST mAb 2947 Rabbit - validated in Figure 4b (p21 signal goes down when its transcription factor p53 is KO'd). Also confirmed by siRNA and KO - not included in the manuscript but available on request.<br>p21 (CP36/CP74) Millipore mAb 05-345 Mouse - validated by KO - not included in the manuscript but available on request.<br>UBE2D2/UbcH5b Novis Bio pAb NPB1-81769 Rabbit - validated in Figure 3e by siRNA.<br>UBE2D3/UbcH5C (D60E2) CST mAb 4330 Rabbit - validated in Figure 3e by siRNA.<br>CENP-C MBL pAb PD030 Guinea Pig - validated in Extended Data Figure 1c-d - stains centromeres.<br>BubR1 Millipore pAb MAB3612 Mouse - validated in Extended Data Figure 1c - stains checkpoint-activated kinetochores.<br>Cyclin B1 (GSN3) Millipore mAb 8A5D12 Mouse - validated in Figure 4b - present in G2/M cells, absent in G1 cells, and in Extended Data Figure 6a-b - mitotic destruction rescued by APC/C inhibition.<br>Cyclin A2 (E399) Abcam mAb Ab32498 Rabbit - validated in Extended Data Figure 6a-b - absent in mitotic cells, rescued by APC/C inhibition.<br>CRBN (D8H3S) CST mAb 71810 Rabbit - validated in Extended Data Figure 7b by siRNA.<br>α-Tubulin (DM1A) Sigma mAb 4394 Rabbit - validated in Extended Data Figure 1c-d - stains spindle microtubules.<br>Cdc20 Proteintech pAb 10252-1-AP Rabbit - validated in Extended Data Figure 6a by siRNA.<br>β-TrCP1 (D13F10) CST mAb 4394 Rabbit - validated in Extended Data Figure 6c by siRNA.<br>GFP (7.1 and 13.1) Roche mAb 11814460001 Mouse - validated in Figure 3b, 6a,c and Extended Data Figure 10a-b - band appears when GFP-tagged transgenes are expressed.<br>p53BP1 Novus Bio pAb NB100-304 Rabbit - validated by KO - not included in the manuscript but available on request. Also labels p53BP1-positive foci in response to DNA damage stimuli, as expected (Extended Data Figure 2d).<br>phospho-p53 (Ser15) CST pAb 9284 Rabbit - validated in Extended Data Figure 2a - induced upon DNA damage. Also validated by KO - not included in the manuscript but available on request.<br>phospho-Chk2 (Thr68) CST pAb 2661 Rabbit - validated in Extended Data Figure 2a - induced upon DNA damage. Also validated by ATM/ATR inhibitor treatment - not included in the manuscript but available on request.<br>Chk2 CST pAb 2662 Rabbit - extensively validated in the literature - not further validated.<br>Arl13b Proteintech pAb 17711-1-AP Rabbit - validated in Extended Data Figure 1b - stains primary cilia.<br>Pericentrin - validated in Extended Data Figure 1a - stains centrosomes (1 in G1 cells, 2 in S/G2/M cells).

Eukaryotic cell lines

Policy information about cell lines and Sex and Gender in Research

Cell line source(s)	Original hTERT-RPE1 (#CRL-4000), HeLa (#CRL-2.2), A375 (#CRL-1619), U2OS (#HTB-96), HEK293T (#CRL-3216), HCT116 (#CCL-247) cell lines were purchased from ATCC.<br>The hTERT-RPE1 p21-GFP cell line was a gift from Alexis R. Barr.<br>Other cell lines generated in this study (e.g. p53 KO hTERT-RPE1 and FUCCI hTERT-RPE1) were derived from these sources.
Authentication	hTERT-RPE1 parental and p53 KO cell lines were STR profiled and authenticated by ATCC.<br>HeLa, HCT116, hTERT-RPE1 FUCCI, and hTERT-RPE1 p21-GFP cell lines were STR profiled and authenticated by NorthGene™.<br>A375, HEK293T and U2OS cell lines purchased from ATCC were not further authenticated.
Mycoplasma contamination	Mycoplasma negative status of cell lines was confirmed using the EZ-PCR Mycoplasma Test Kit with internal control (K1-0210, Geneflow).
Commonly misidentified lines<br>(See ICLAC register)	The cell lines used in our studies are not on the list as commonly misidentified lines.
