# Supplementary material for: MDM2 functions as a timer reporting the length of mitosis
Source: Nat Cell Biol. 2025 Jan 9;27(2):262–72. doi: 10.1038/s41556-024-01592-8 (PMC11821534; doi:10.1038/s41556-024-01592-8)

Source Data - Fig. 2

Fig. 2b

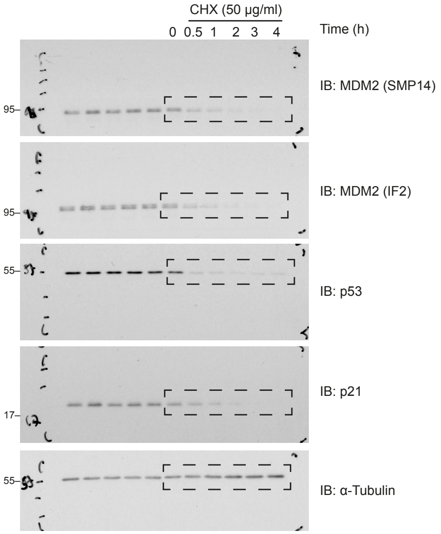

Fig. 2c

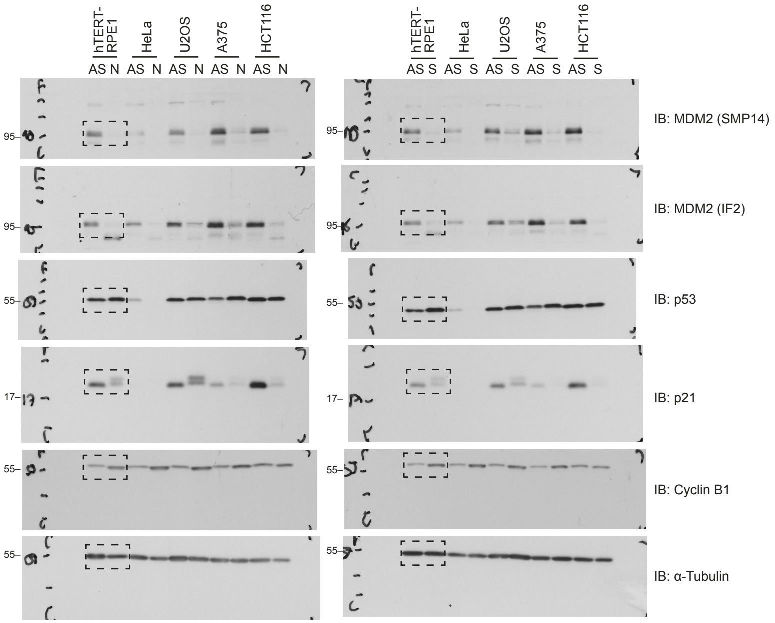

Fig. 2e

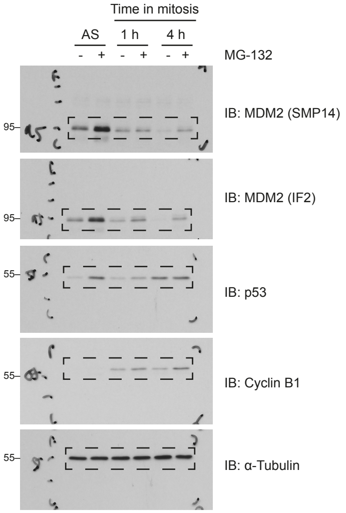

Fig. 2g

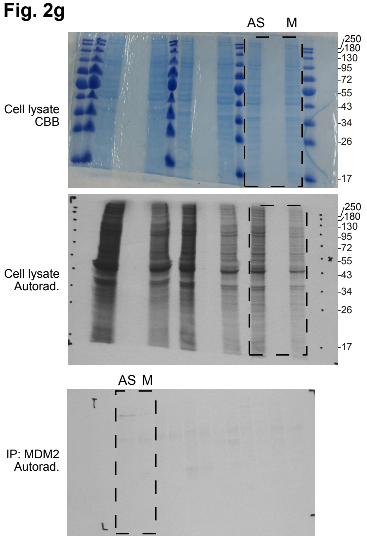

Source Data - Fig. 3

Fig. 3b

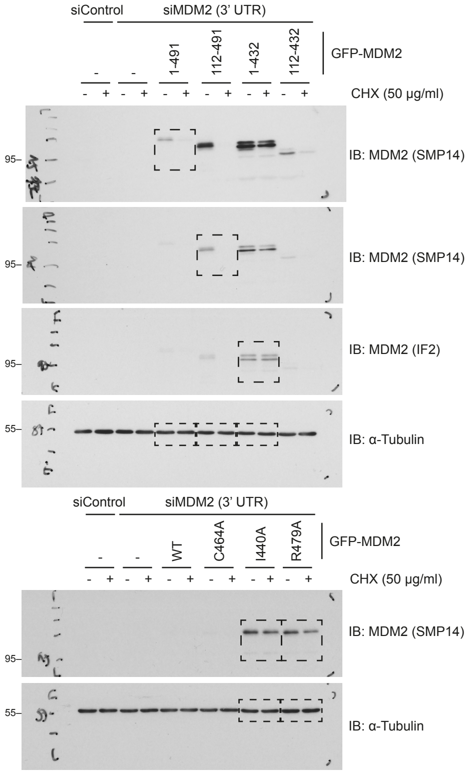

Fig. 3c

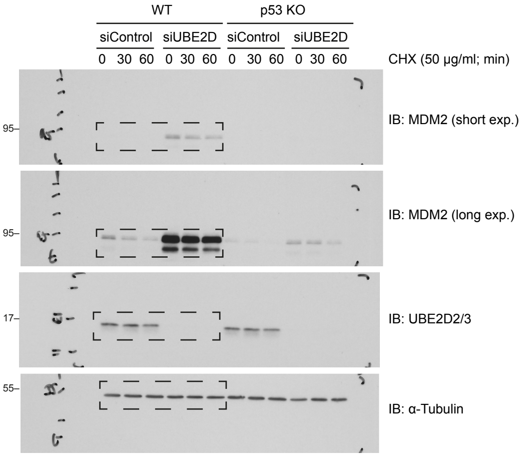

Fig. 3d

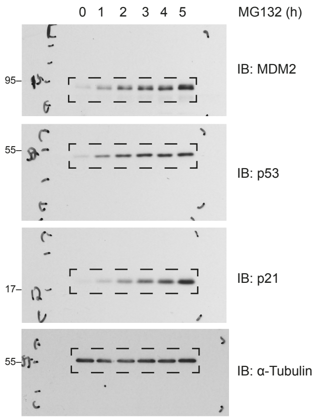

Fig. 3e

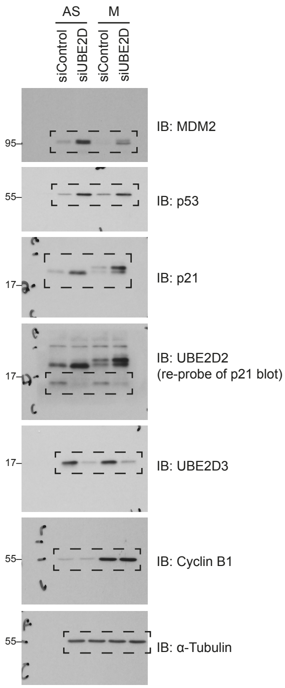

Source Data - Fig. 4

Fig. 4b

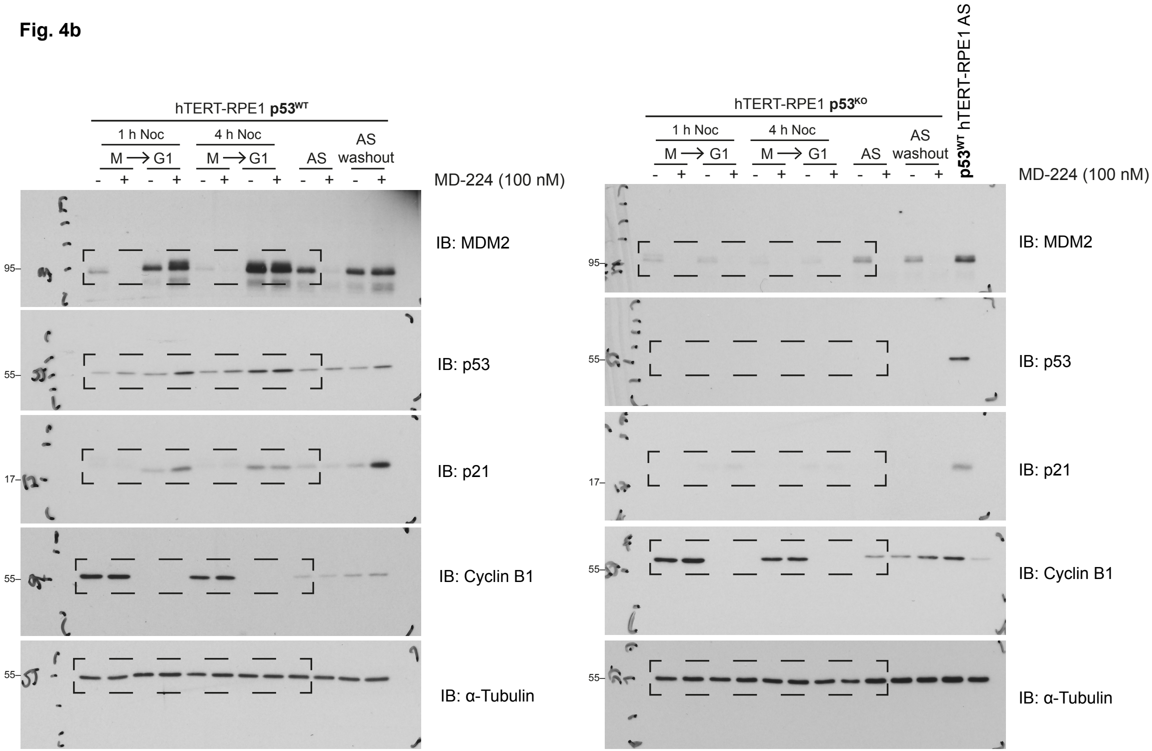

Source Data - Fig. 5

Fig. 5g

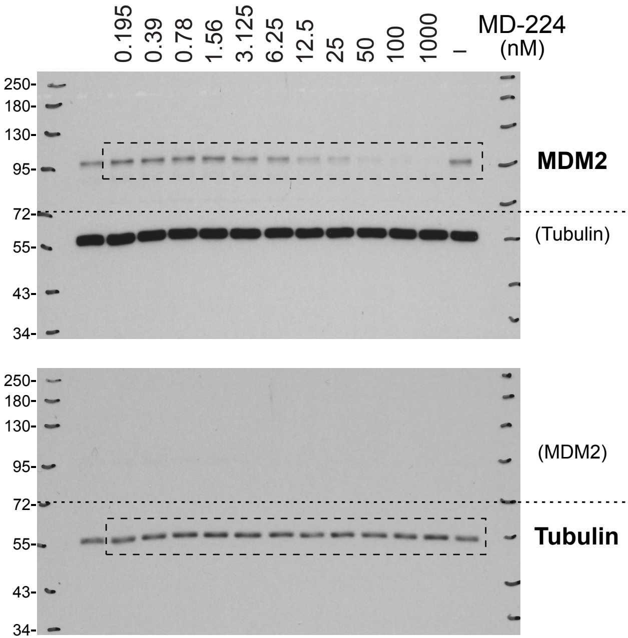

Source Data - Fig. 6

Fig. 6a

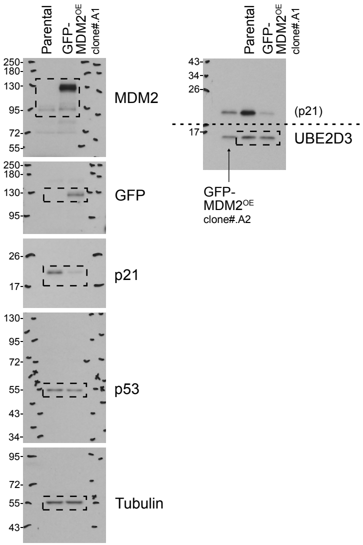

Fig. 6c

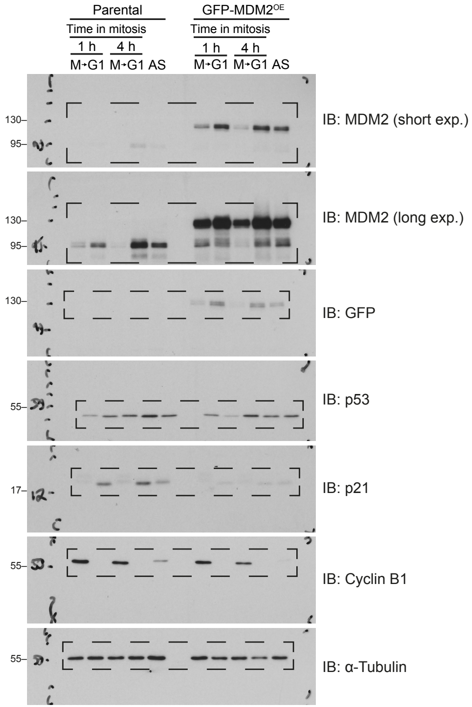

Source Data - Extended Data Fig. 2

Extended Data Fig. 2a

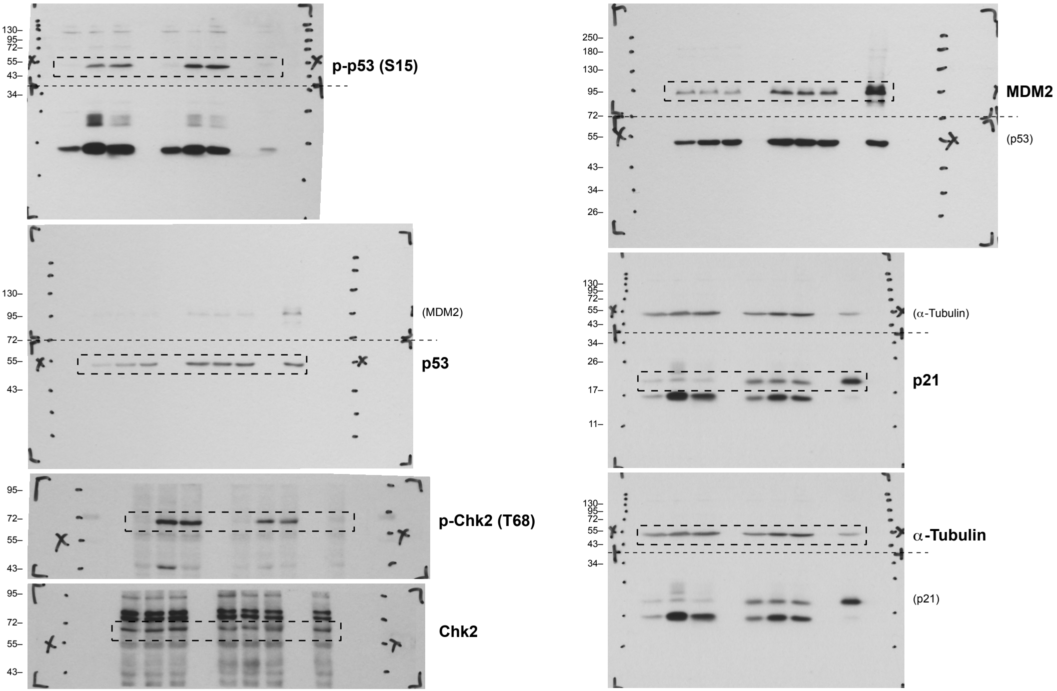

Source Data - Extended Data Fig. 3

Extended Data Fig. 3a

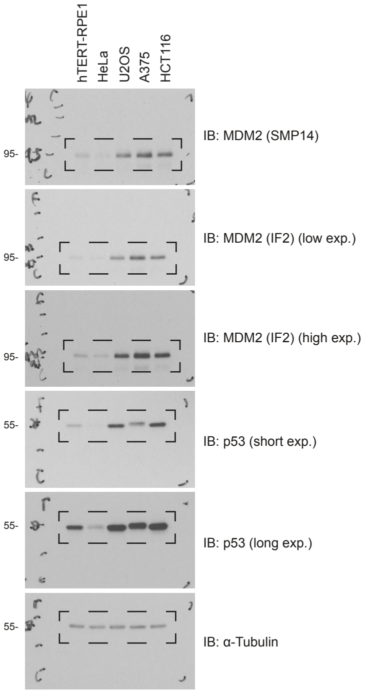

Extended Data Fig. 3c

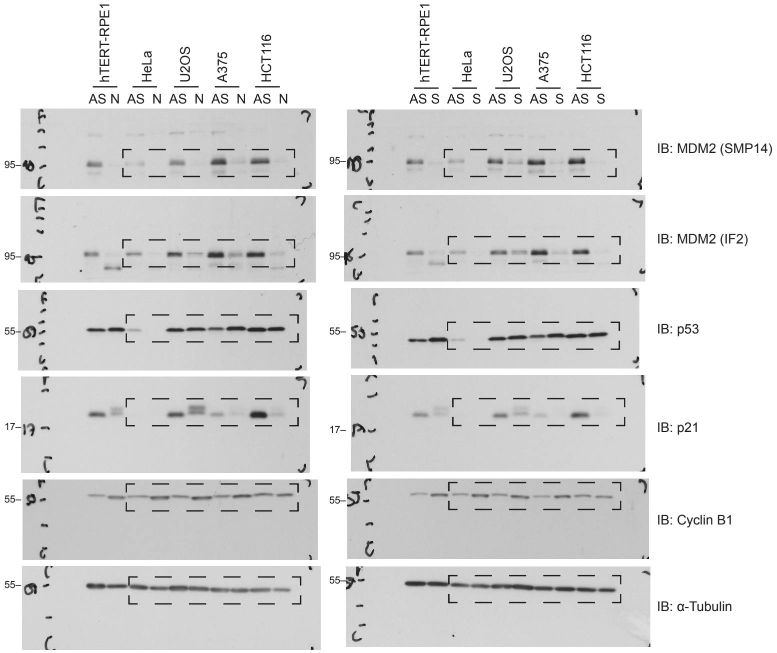

Source Data - Extended Data Fig. 4

Extended Data Fig. 4

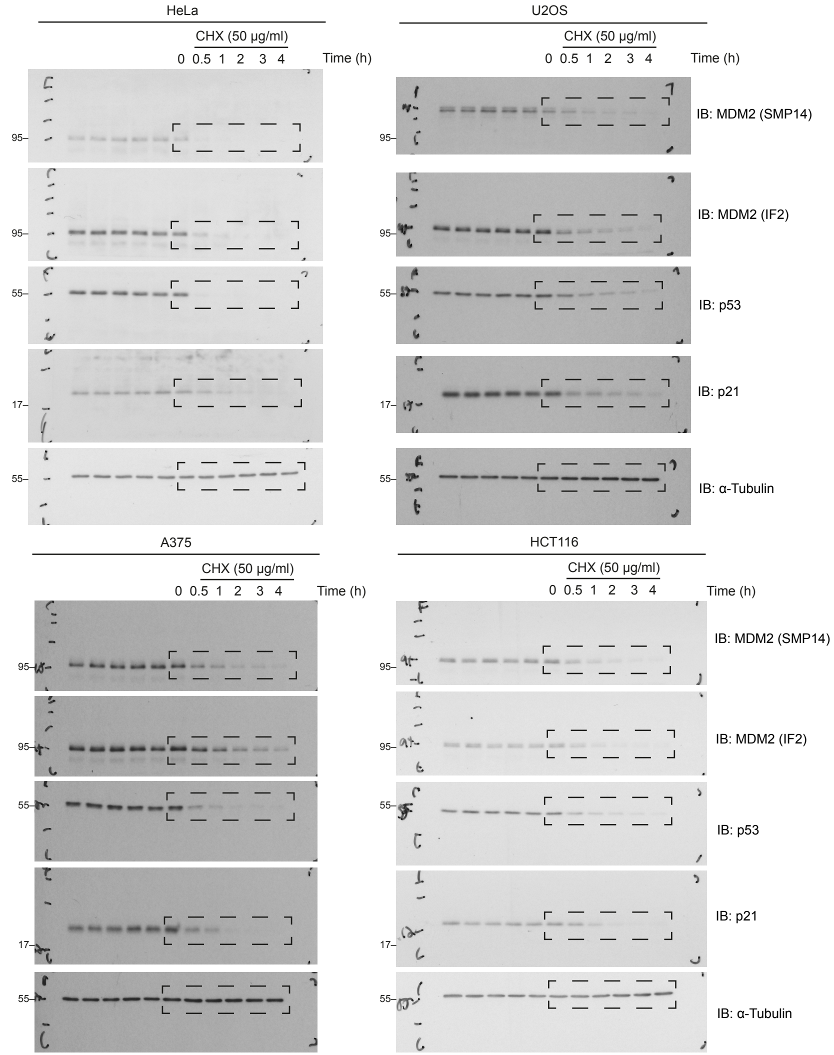

Source Data - Extended Data Fig. 5

Extended Data Fig. 5d

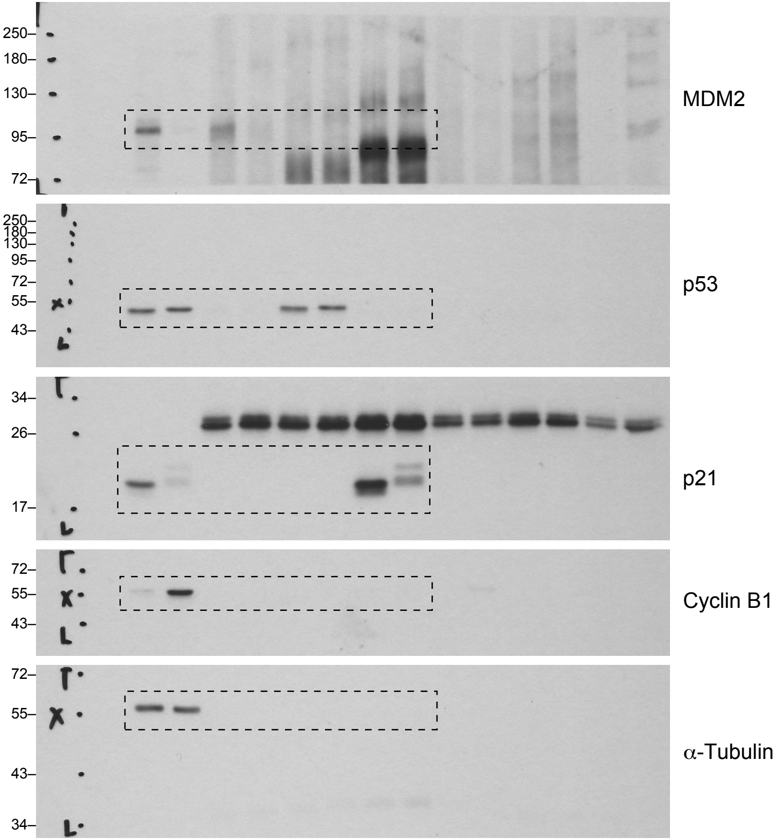

Source Data - Extended Data Fig. 6

Extended Data Fig. 6a

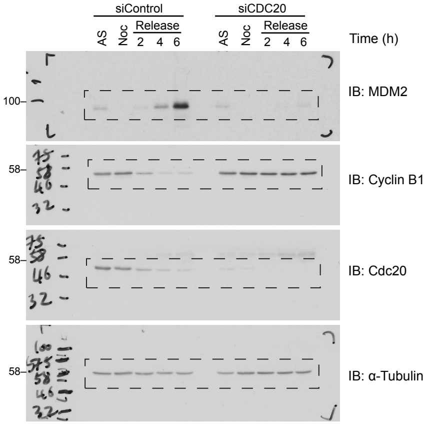

Extended Data Fig. 6b

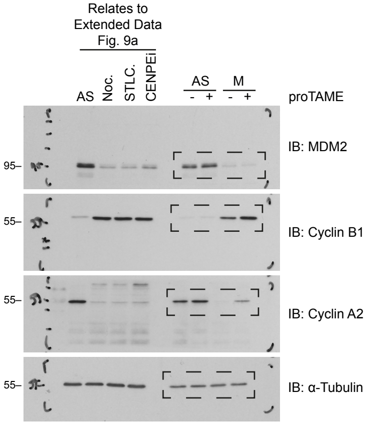

Extended Data Fig. 6c

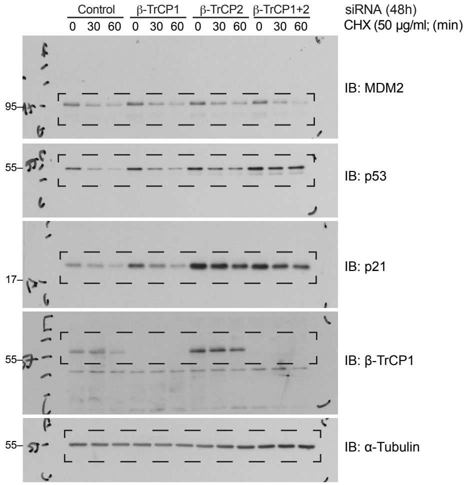

Source Data - Extended Data Fig. 7

Extended Data Fig. 7b

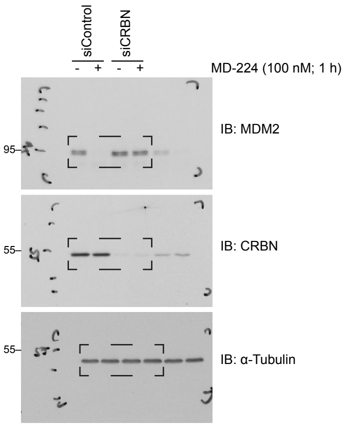

Extended Data Fig. 7c

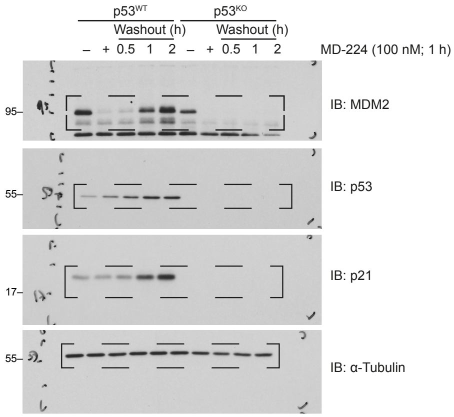

Extended Data Fig. 7d

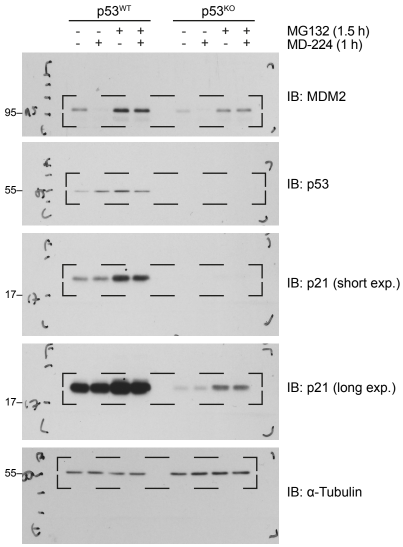

Extended Data Fig. 7e

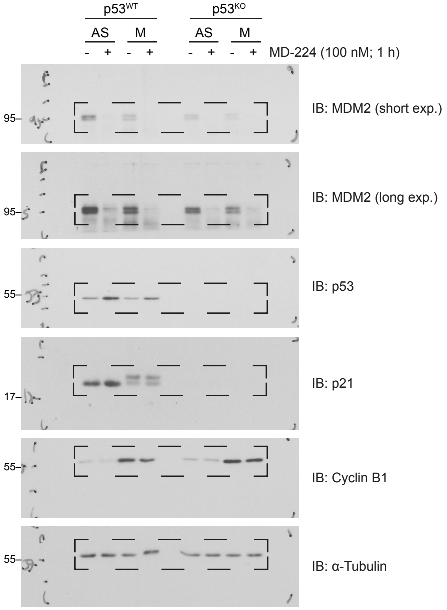

* Mitotic cells (1 h Noc. arrest)

Extended Data Fig. 7f

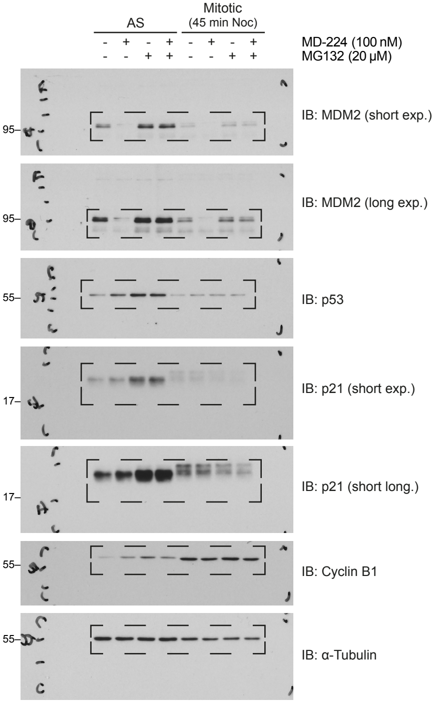

Source Data - Extended Data Fig. 9

Extended Data Fig. 9a

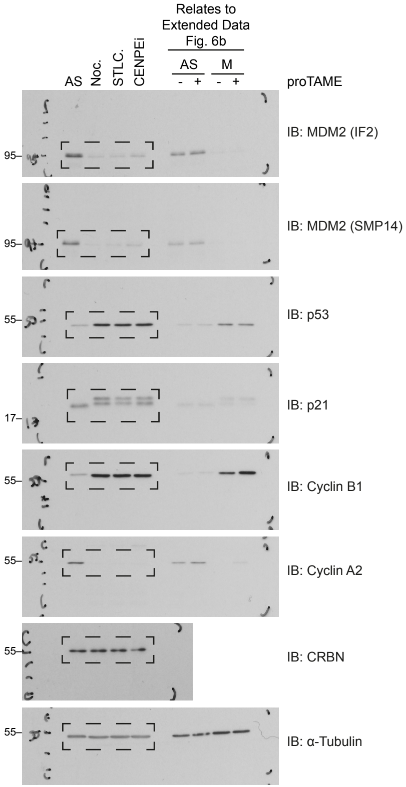

Extended Data Fig. 9c

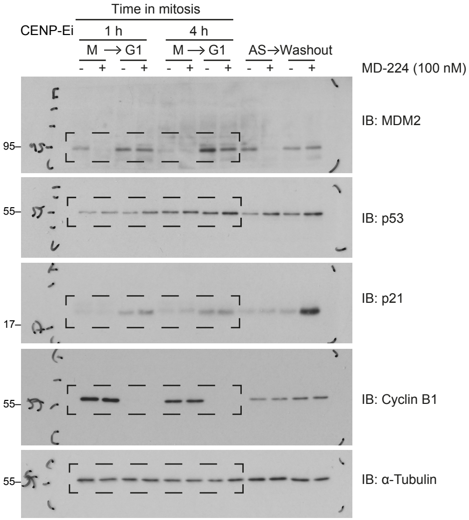

Source Data - Extended Data Fig. 10

Extended Data Fig. 10a

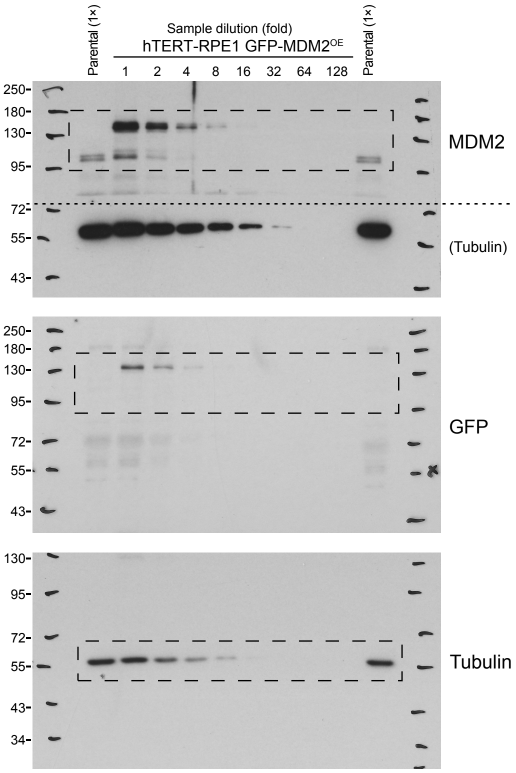

Extended Data Fig. 10b

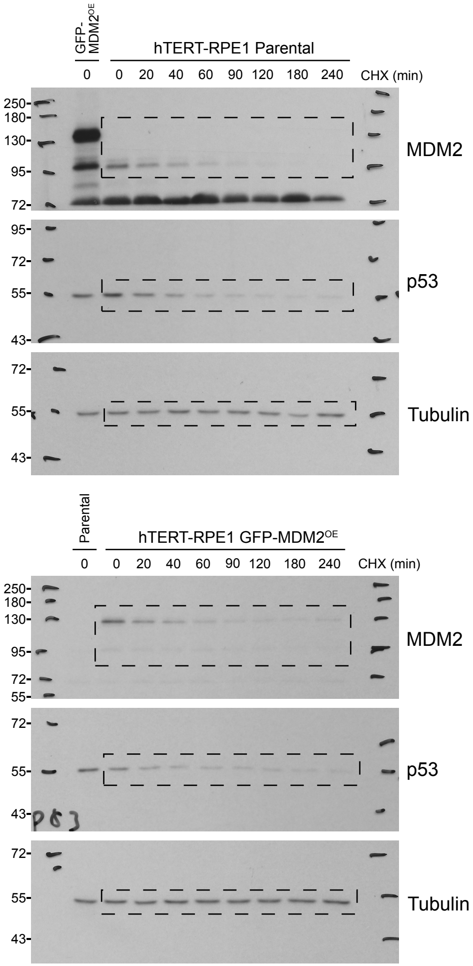

Supplement: Supplementary file 5 — Unprocessed western blots and gels. [file 41556_2024_1592_MOESM5_ESM.pdf]
